# Advancing equity research in the quality of and access to health care in a post‐affirmative action era

**DOI:** 10.1111/1475-6773.14249

**Published:** 2023-11-28

**Authors:** Michael K. Ong, Keith C. Norris

**Affiliations:** ^1^ Division of General Internal Medicine & Health Services Research, Department of Medicine, David Geffen School of Medicine University of California Los Angeles Los Angeles California USA; ^2^ Department of Health Policy and Management, Fielding School of Public Health University of California Los Angeles Los Angeles California USA; ^3^ Center for the Study of Healthcare Innovation, Implementation, and Policy, Veteran Affairs Greater Los Angeles Healthcare System Los Angeles California USA; ^4^ Office of Equity, Diversity, Inclusion, Department of Medicine, David Geffen School of Medicine University of California Los Angeles Los Angeles California USA

*It is much more important to know what sort of a patient has a disease, than what sort of disease a patient has*.

William Osler

## 1 INTRODUCTION

Despite powerful narratives echoing a purported existence of meritocracy in our society, major racial and ethnic disparities in health and healthcare have persisted for hundreds of years. Yet a clear understanding of structural societal inequities and not innate group differences underlying the racial disparities in health was articulated nearly 200 years ago by Dr. James McCune.^1^ His position was reified in the findings from “The Philadelphia Negro,” an elegant sociological and epidemiological study by Dr. W.E.B. Du Bois in the late 1800s.^2^ However, their voices were largely ignored because voices of Native and Black Americans have been devalued in America to maintain a myth of racial inferiority.

It was not until the 1985 Report of the Secretary's Task Force on Black and Minority Health, led by Dr. Margaret Heckler, that the nation could accept such disparities as a problem worthy of consideration. Soon thereafter, the 2003 Institute of Medicine report entitled “Unequal Treatment: Confronting Racial and Ethnic Disparities in Health Care” sought to not only codify racial and ethnic group differences in health outcomes, but emphasized these were due to how people were treated and not due to innate attributes. It also identified how this mistreatment led to differences in the quality of and access to health care.

The emerging focus on the quality of and access to health care at the turn of the 21st century was embraced by the Agency for Healthcare Research and Quality (AHRQ) who in 2003 started to formally track disparities in health care delivery as it relates to racial and socioeconomic factors in priority populations. This formal tracking of disparities played a critical role in developing the evidence base for health systems to improve and advance their ability to provide the best care to all of us. Spurred by the Coronavirus 2019 (COVID‐19) pandemic and the 2020 murder of George Floyd, the US health care system faced a social and health equity justice movement. This movement emphasized the role of racism and the inequitable distribution of the social determinants of health (SDoH), the broad set of forces and systems shaping the conditions of daily life, as major driving forces in health disparities and major root causes in the racial and ethnic disparities in the access to and quality of health care. In 2022, AHRQ sponsored a Health Equity Summit to bring together multiple working groups from the 2021 *AHRQ Equity Agenda and Action Plan* that was created to help guide priorities to advance health equity. This commentary addresses two interrelated articles, written by Chisholm et al.^3^ and Jindal et al.^4^ that describe the findings from AHRQ's 2022 Health Equity Summit and highlight critical stages and key action steps in the future of equitable health care delivery.

## 2 AHRQ AND ITS FUTURE HEALTH EQUITY AGENDA

Chisholm et al.^3^ describe six priority research themes for health care systems to promote health equity and population health, while strengthening access to care: (1) institutional leadership, culture, and workforce; (2) data‐driven, culturally tailored care; (3) health equity‐targeted performance incentives; (4) health equity‐informed approaches to health system consolidation and access; (5) whole‐person care; and (6) whole community investment. They also propose complementary action plans as follows: (1) publish white papers and toolkits on evidence‐informed workforce; (2) support policies that require patient race ethnicity and language data collection by providers and payers; (3) develop toolkits to assist health care organizations integrate equity metrics into their performance management systems as well as develop publicly available equity‐focused evidence‐based quality indicators; (4) support the development of geographic information systems to track changes in the health care access, quality, and equity arising from consolidation and/or policy changes; (5) accelerate research on health outcomes linked to social needs; and (6) develop a new health equity research funding models with funding allocated directly to community organizations working in partnership with universities or health care organizations using the Small Business Innovation Research model. The last theme of meaningful whole community engagement, investment, and partnerships with the local communities that health care systems serve is in many ways the action with the greatest potential to transform people and policies to begin truly addressing the root causes of racial and ethnic disparities in the access to and quality of health care and more.^5^

Jindal et al.^4^ highlight the need for AHRQ to leverage partnerships with other federal agencies and develop public–private partnerships in pursuit of undoing systemic barriers and inequities, and describes five dimensions for a health equity research agenda that focuses on improving access to care: (1) approachability, (2) acceptability, (3) availability and accommodation, (4) affordability, and (5) appropriateness. Complementary actions steps include: (1) collaboratively develop and evaluate an evidence‐based trustworthy anti‐racism toolkit for health systems; (2) champion the use of evidence‐based tools for addressing structural racism; (3) fund collaborative research to identify causes of burnout with a lens on intersectionality and mistreatment; (4) fund research to examine the costs of maintaining the flexibility for state Medicaid programs, identify coverage improvements to the Affordable Care Act, and find how to eliminate discriminatory structural barriers to accessing health insurance; and (5) support research on patient‐centered decision‐making tools that integrate intersectionality with a health equity lens as well as the de‐implementation of low‐value care and elimination of individual‐level race‐based algorithms. They note, “progress will be made when there is common acceptance that inequities in access, health, and disease arise from a disease process rooted in social experience and infrastructure rather than DNA”.

Together, these articles reflect the importance of how addressing inequities in access to and quality of health care must be done with consideration of systemic racism and intersectionality, and emphasize the importance of high‐quality research to inform equitable health care delivery.

These outcomes and recommendations from the AHRQ's 2022 Health Equity Summit help to more optimally position AHRQ to address inequities in access to and quality of health care, created and once again perpetuated by legal decisions during this time of potential new limits in tools we have available to achieve equity. The importance of a focus on the SDoH as a pathway through which racism works is critical given the potential implications of the new Supreme Court rulings, which state that universities can consider “an applicant's discussion of how race affected his or her life, be it through discrimination, inspiration, or otherwise.”^6^, ^7^ In other words, race cannot be used as a criterion for admission alone, yet, an applicant can discuss how they may have been impacted by race (or racism). A similar approach may need to be undertaken by AHRQ if there is extrapolation of university admission policy recommendations to actions to promote equity in access to and quality of health care.^11^

## 3 HEALTH EQUITY IN A POST‐AFFIRMATIVE ACTION ERA

AHRQ's Health Equity Summit was prescient in its timing, preceded two major landmark events with major implications for the health equity landscape—the recent Supreme Court rulings (Students for Fair Admissions, Inc. v. President and Fellows of Harvard College and Students for Fair Admissions, Inc. v. University of North Carolina; 303 Creative LLC v. Elenis), which have ended affirmative action in college admissions and yet allowed discrimination based on free expression despite public accommodation laws. These rulings do not mean that racial and ethnic or other identity‐based inequities no longer exist, particularly with regard to health. However, these rulings limit the approaches that can be used to address health inequities, particularly those focused on health workforce composition or regulations requiring equal provision of service. We will collectively need to innovate to ensure that health systems have the appropriate research findings and data to implement requisite solutions to reduce health disparities and improve health equity. This is why a focus by AHRQ explicitly on equity in access to and quality of health care will be critical, as AHRQ can be the driver for necessary health equity innovation through its research agenda and funding priorities.

Some have argued that health care organizations are not well equipped to take on many of the root causes of health disparities such as inequities in the allocation of social determinants of health (SDoH).^8^ Many patients interfacing with health care organizations are suffering from systematic indifference to the inequitable distribution of health and life‐affirming resources. The inability of state and federal agencies to approve the necessary policy changes to achieve equity in the distribution of resources creates a dilemma for health care organizations who ultimately cannot afford to ignore the SDoH, as they will continue to have long‐term effects on patient outcomes, health care utilization and costs. Indeed, Laviest et al. have estimated the direct and indirect economic burden of health disparities to exceed $400 billion a year.^9^ As noted by both Chisholm et al.^3^ and Jindal et al.^4^ AHRQ can lead the way to determine best ways for health care organizations to address this dilemma by partnering with social service organizations and more, while working with other federal agencies and private–public partnerships.

However, to truly reverse many of the structural and longstanding issues that underpin health inequities, AHRQ will need to directly partner more closely with affected communities.^3^, ^4^, ^10^ Direct partnerships will accelerate the community buy‐in needed for investigator teams to be successful with the work they will be conducting with the health care organizations that provide care to these communities, and subsequently allow AHRQ to truly achieve success with its health equity agenda. Some of these partnerships are slowly beginning to occur more broadly as universities, hospitals and other large, health organization become “Anchor Institutions” that commit major financial, human and intellectual resources to address social challenges, understanding that their future is inextricably linked to the community outside their walls.^11^

## 4 CONCLUSION

Ensuring health equity is critical for all of us in the care we receive from health care delivery systems, especially the components that affect our ability to access care. Implementation of these proposed approaches for AHRQ to infuse its research agenda would be important steps toward achieving health equity.

## FUNDING INFORMATION

Dr. Ong receives relevant funding from the Agency for Healthcare Research and Quality (K12 HS026407) and the Department of Veteran Affairs Health Services Research & Development (CIN 13‐417). Dr. Norris receives relevant funding from the National Institutes of Health (P50MD017366, P30AG021684, UL1TR000124).
